# Overview of distinct 8-oxoguanine profiles of messenger RNA in normal and senescent cancer cells

**DOI:** 10.3389/fcell.2025.1443888

**Published:** 2025-02-28

**Authors:** Jingwen Huang, Yu Lin, Yingying Zhao, Lingbo Wei

**Affiliations:** ^1^ Qingdao TCM Geriatrics Diagnosis and Treatment Center, Department of Geriatrics, Qingdao Hiser Hospital Affiliated of Qingdao University (Qingdao Traditional Chinese Medicine Hospital), Qingdao, China; ^2^ Department of Gastroenterology, Southern Medical University Hospital of Integrative Chinese and Western Medicine, Southern Medical University, Guangzhou, China

**Keywords:** 8-oxoguanine, senescence, RNA methylation, MeRIP-seq, cancer cell

## Abstract

**Background:**

Cellular senescence plays a key role in the development of cancer, but the underlying mechanisms are unknown. Recently, several recent studies have shown that RNA methylation is closely related to cancer cell aging. 8-Oxoguanine (o^8^G) is an important and widely distributed methylation modification whose role in cancer cell senescence is far from elucidated.

**Methods:**

In this study, senescent cancer cell models (CaCO_2_ cells) were constructed by knocking down the ADAR1 gene. RNA immunoprecipitation sequencing was used to identify the o^8^G peaks on messenger RNA (mRNA) of normal CaCO_2_ cells and senescent CaCO_2_ cells, and the distribution characteristics of mRNA o^8^G modification were identified. Further bioinformatics analysis of the sequencing data was performed to preliminarily elucidate the potential function of the o^8^G-modified mRNA.

**Results:**

There were significant differences in mRNA o^8^G modification distribution between normal and senescent CaCO_2_ cells. It is suggested that o^8^G modification may play a key role in inducing cancer cells or promoting cancer cell senescence. Gene ontology (GO) enrichment analysis showed that the mRNAs modified by o^8^G were enriched in Cellular component organization or biogenesis, Focal adhesion, and RNA binding. Kyoto Encyclopedia of Genes and Genomes (KEGG) pathway analysis showed that the genes modified by o^8^G are concentrated in Focal adhesion signaling pathway, Small cell lung cancer signaling pathway and Proteoglycans in cancer signaling pathway.

**Conclusion:**

This study preliminarily revealed the different distribution patterns of o^8^G modification between normal CaCO_2_ cells and senescent CaCO_2_ cells. Our study established the link between o^8^G modification and cancer cell senescence, which provides a new insight into the mechanism of cancer cell senescence and a potential therapeutic target for subsequent cancer treatment.

## Introduction

Cellular senescence is a state of growth arrest induced by severe injury and stress, accompanied by a hypersecretory phenotype, morphological changes, and telomere dysfunction (Wang et al., 2022). Several inducing conditions, such as activated oncogenes, cytokines, reactive oxygen species, DNA damage, and nucleotide depletion, can trigger cellular senescence (Calcinotto et al., 2019; Chandrasekaran et al., 2017; Herranz and Gil, 2018; van Deursen, 2014). In cancer cells, senescence exhibits two distinct functions of anti-tumor and pro-tumor. For example, senescence can induce growth arrest in cancer cells, but it can also promote cancer progression through senescence-mediated tumorigenesis immune evasion (Schmitt et al., 2022).

Disorders of epigenetic regulation are an important feature of cellular senescence (López-Otín et al., 2013), and most of the epigenetic modifications are reversible relative to the genetic regulation represented by DNA sequences, suggesting that epigenetic regulation can be intervened to delay aging and treat aging-related diseases. Therefore, through the study of epigenetic changes in the aging process, we can better understand how to delay aging through external means. Epigenetic alterations play an important regulatory role in cellular senescence (Sen et al., 2016). However, most studies have focused on, for example, histone modifications (Baell et al., 2018) and DNA methylation (Xie et al., 2018). However, as an important epigenetic regulator, the role of RNA modification in the cellular senescence of cancer cells is still poorly understood, and only a few studies have explored the role of RNA modification in cancer cell senescence. METTL3 plays a key role in replicating senescence of colorectal cancer (CRC) cells, and inhibition of METTL3 or targeted inhibition of CDKN2B methylation can effectively inhibit CRC senescence (Chen et al., 2024). In HeLa cells, METTL3/14 and ADAR1 synergistically increase the translation of p21 in response to oxidative stress, leading to cellular senescence (Li et al., 2017). In addition, there is a study that explores the role of m1 A in cellular senescence. Knockdown of Alkbh3 in NSCLC cells leads to senescence induction and cell cycle arrest, followed by increased expression of cell cycle arrest proteins p27 and p21 (Tasaki et al., 2011).

8-oxoguanine (o^8^G) RNA modification is a new RNA modification that has attracted extensive attention in the field of molecular biology and epitranscriptomics in recent years. This modification involves the guanine nucleotide in the RNA molecule being attacked by reactive oxygen species (ROS) under specific conditions, which in turn converts to o^8^G. While 8-oxoguanine (8-oxo-dG) in DNA has been extensively studied, o^8^G modifications in RNA are more common, as RNA is more susceptible to oxidative damage. This difference may be attributed to the single-stranded nature of RNA and its more dynamic distribution in cells (Hahm et al., 2022).

Recent studies have shown that o^8^G modification is not just a consequence of oxidative damage, it may also play a role in regulating gene expression. For example, it has been found that position-specific o^8^G modifications can occur in the seed region of miRNAs and modulate mRNA stability and translation through specific base pairing (Seok et al., 2020). Determining the distribution of o^8^G within the transcriptome can deepen our understanding of o^8^G.

Methylated RNA immunoprecipitation sequencing (MeRIP-seq) is a commonly used technique for detecting o^8^G modifications. This method breaks down purified total RNA or mRNA into 100–150 nt RNA fragments; Subsequently, RNA fragments were co-incubated with o^8^G-modified antibodies, and the o^8^G-modified RNA fragments were enriched with antibodies for library construction and sequencing. Finally, the o^8^G-modified transcriptome region was obtained by comparison with the control library co-incubated with the o^8^G-modified antibody.

ADAR1 is an important regulator of aging, with knockdown of ADAR1 at the cellular level leading to cellular senescence, and overexpression of ADAR1 prevents senescence from occurring (Hao et al., 2022). In this study, we constructed an ADAR1-deficient colorectal cancer cell line to induce senescence in colorectal cancer cells. MeRIP-seq was performed on normal CaCO_2_ cells and senescent CaCO_2_ cells induced by ADR1 knockdown, and o^8^G-specific analysis and in-depth bioinformatics analysis were performed on o^8^G mRNA.

The results showed that there were significant differences in the number and distribution of o^8^G between the two groups. The number of o^8^G methylation peaks in normal CaCO_2_ cells was much higher than that in ADAR1 knockdown cells, and the distribution of o^8^G methylation peaks was significantly different, involving all chromosomes. Further bioinformatics analysis showed that the two groups of cells with different degrees of o^8^G methylation could cause significantly different changes in cell function. Our study preliminarily suggests an association between CRC and o^8^G modification in mRNA and predicts potential functional changes due to differences in o^8^G modification in mRNA. These results provide a solid foundation for further understanding the mechanism of cancer cell senescence from the perspective of o^8^G modification.

## Materials and methods

### Establishment of senescent colorectal cancer cell line

ADAR1 is an important regulator of aging, knockdown of ADAR1 can lead to cellular senescence, and overexpression of ADAR1 can prevent senescence from occurring. In the present study, we constructed ADAR1 deletion-induced senescence in colorectal cancer cells. The ADAR1 knockdown lentivirus construction system was obtained from Obio Company (Shanghai, China). CaCO_2_ cells were stably transfected with ADAR1 knockdown lentivirus (shADAR1) and a corresponding control (shNC) with an anti-puromycin plasmid. The stably transfected CaCO_2_ cell line was treated with puromycin (4 μg/mL) for 14 days for selection. The sequence of shRNA is GCC​CAC​TGT​TAT​CTT​CAC​TTT.

### RNA extraction and fragmentation

In this study, we extracted total RNA using Trizol reagent (Invitrogen, CA, United States) and reduced rRNA content using Ribo-Zero rRNA removal kit (Illumina, Inc., CA, United States). The quality of RNA was assessed using the OD260/OD280 ratio. When OD260/OD280 values are in the range of 1.8–2.1, RNA purity is considered to meet the standard, and RNA extracted from all samples in this study meets this standard. In this study, we extracted total RNA using Trizol reagent (Invitrogen, CA, United States) and reduced rRNA content using Ribo-Zero rRNA removal kit (Illumina, Inc., CA, United States). The quality of RNA was assessed using the OD260/OD280 ratio. When OD260/OD280 values are in the range of 1.8–2.1, RNA purity is considered to meet the standard, and RNA extracted from all samples in this study meets this standard.

### Library construction and sequencing

Methylated RNA immunoprecipitation sequencing (MeRIP-seq) refers to the procedure previously reported in the literature (Lin et al., 2023). Three biological repeats of immunoprecipitation of o^8^G-containing RNA were performed under each condition and ribosomal RNA (rRNA) was removed from the sample using the GenSeq^®^ rRNA Removal Kit (GenSeq, Inc.) according to the manufacturer’s instructions. After the samples were qualified, o^8^G RIP kit from GenSeq was used for o^8^G-IP reaction. The process is described as follows: RNA is randomly fragmented into fragments of about 200 nt. Protein A/G magnetic beads and Anti-o^8^G antibody (QED Bioscience, Cat# 12501) were incubated at room temperature for 1 h to bind the antibodies to the magnetic beads. The RNA fragments were then incubated with an antibody bound with magnetic beads at 4°C for 4 h to bind the RNA to the antibody. After incubation, the RNA/antibody complex is cleaned several times. The captured RNA is then eluted from the complex and purified. RNA libraries of purified products were constructed using the GenSeq^®^ Low Input Whole RNA Library Prep Kit (GenSeq, Inc.) kit, and quality control was performed using the Agilent 2100 bioanalyzer. Finally, the library was sequenced by illumina 6000 with 2 × 150 bases in high throughput.

### Identifications and analysis of 8-oxoguanine peaks

Raw reads (Raw Data) are generated after sequencing, image analysis, base recognition, and quality control on an Illumina novaseq 6000 sequenator. Start with the Q30 for quality control. Q30 > 80% indicates good sequencing quality. Then, use cutadapt software (v1.9.3) (Kechin et al., 2017) to remove the low-quality reads and obtain high-quality clean reads. The clean reads of all samples were matched to the reference genome using Hisat2 (Kim et al., 2015) software (v2.0.4). The MACS (Zhang et al., 2008) software was then used to identify the o^8^G-modified gene in each sample. Gene identification of differentially o^8^G modifications was performed using diffReps (Shen et al., 2013) software. Use your own program to screen for peaks located on the exon of the mRNA and annotate accordingly.

### Statistical analysis

The o^8^G peaks on the mRNA of the samples in each group were combined to obtain the o^8^G peaks of each group. Use the Bed Tool software to find the common peaks between the two groups. The 50 bp sequences on either side of the apex of the o^8^G peak were scanned using DRAME software (Bailey, 2011) to find meaningful motifs. The E value of the motif is calculated as the enriched P value multiplied by the number of candidate motif for the test, and the enriched P value is calculated using Fisher’s exact test to enrich the motif in the positive sequence. The lower the E value, the higher the confidence level. Methylation fold enrichment (FE) of each mRNA in the sample was collected and log2 converted. The log FE value is used for clustering in the heatmap. The mRNA region in which the o^8^G peak is located in each sample was calculated according to the published method and the results were plotted as a pie chart.

### Bioinformatics analysis

**FIGURE 1:**
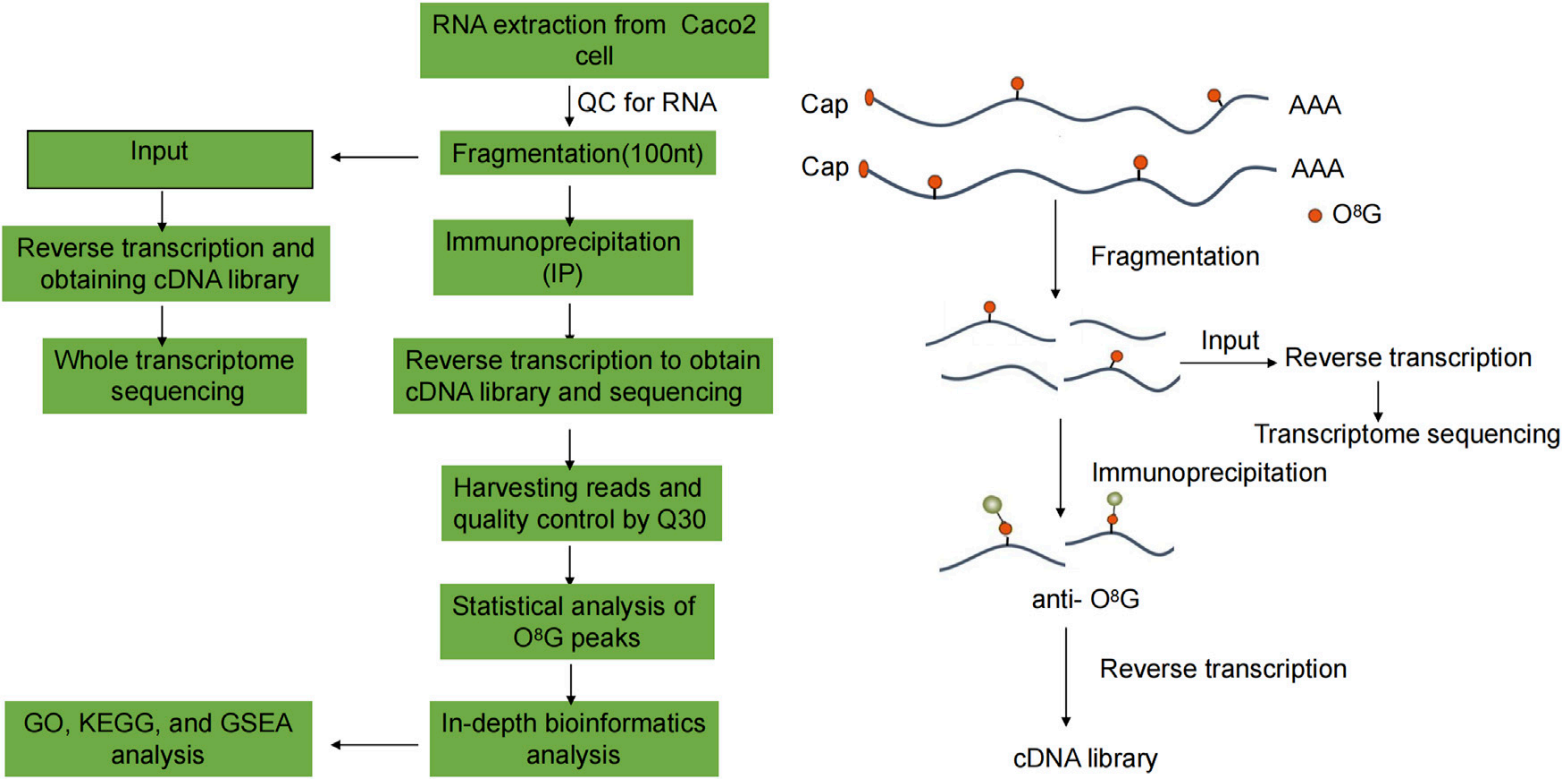
Flowchart of the study.

Functional analysis (http://www.geneontology.org) of differentially o^8^G-modified coding genes was performed using Gene Ontology (GO) to annotate and speculate on the function of these differentially methylated genes. Pathway analysis of differentially o^8^G-modified coding genes was performed using the Kyoto Encyclopedia of Genes and Genomes (KEGG) (https://david.ncifcrf.gov/) to annotate and speculate on the pathways in which they may be involved. In addition, we used the fold change in enrichment intensity of the two groups of samples in the MeRIP-seq experiment to sequence the signals of all coding genes. We chose FDR < 0.25 as the screening criteria. The technology roadmap is shown in Figure 1.

## Result

### Construction of senescent cancer cells by knockdown ADAR1

SAβ-Gal is a commonly used marker of cellular senescence. SA-β-Gal (Senescence-Associated β-Galactosidase) is a β-galactosidase that exhibits increased activity during cellular senescence. By measuring the activity level of SA-β-Gal in cells, the degree of senescence of cells can be assessed. The results of SA-β-Gal showed that CaCO_2_ cells senescent compared with normal cells after knockdown of ADAR1, as shown in Supplementary Figure S1.

### General features of o^8^G methylation in CaCO_2_ cells and sh-ADAR1 CaCO_2_ cells

**FIGURE 2:**
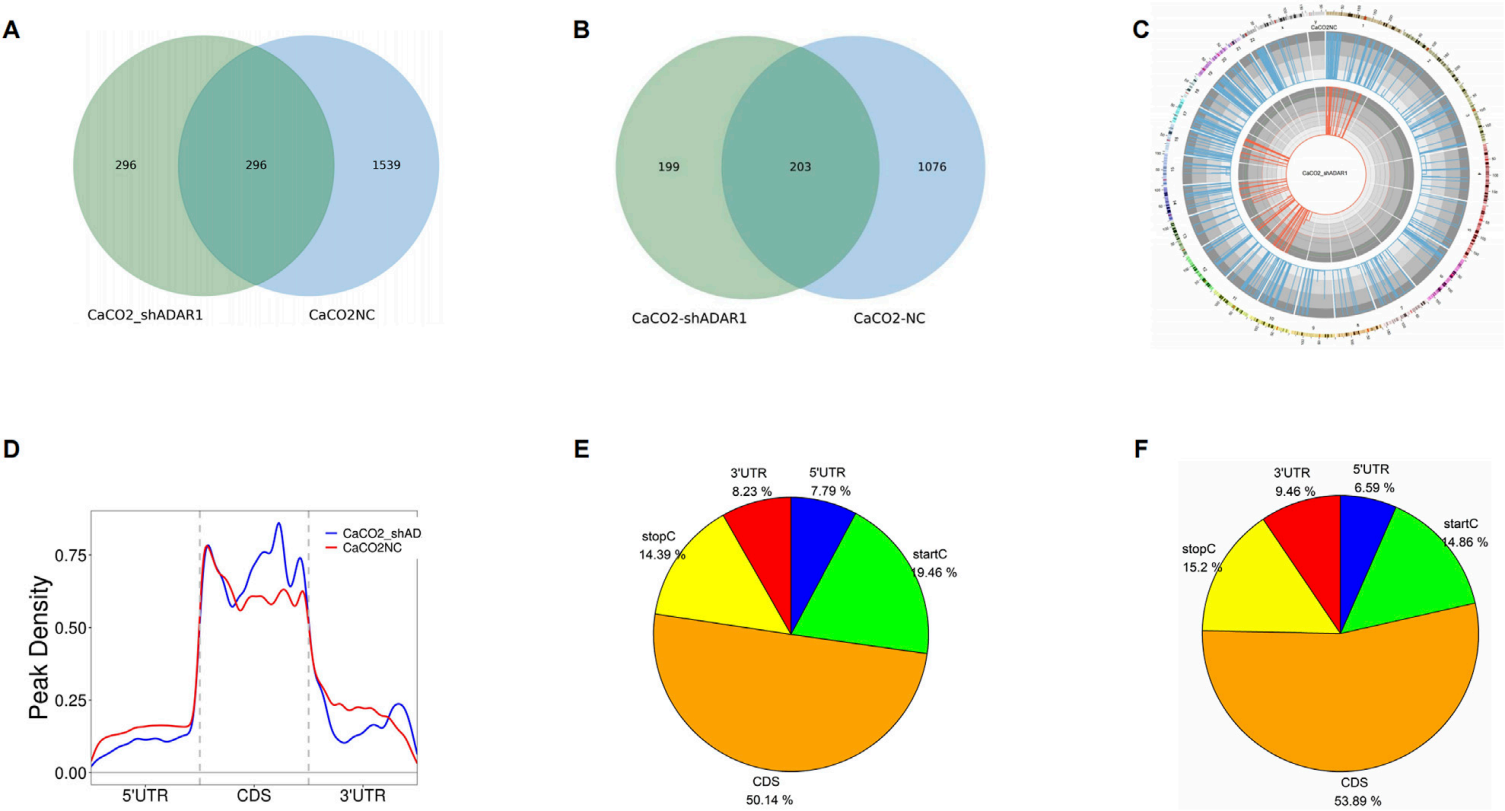
Characteristics of o^8^G peaks in CaCO_2_ cell and sh-ADAR1 CaCO_2_ cell. **(A)** Venn diagram of o^8^G peaks in CaCO_2_ cell and sh-ADAR1 CaCO_2_ cell. **(B)** Venn diagram of o^8^G genes in CaCO_2_ cell and sh-ADAR1 CaCO_2_ cell. **(C)** Visualization of o^8^G at the chromosome level in CaCO_2_ cell and sh-ADAR1 CaCO_2_ cell. **(D)** Accumulation of the region of average o^8^G peaks along all transcripts in CaCO_2_ cell and sh-ADAR1 CaCO_2_ cell. **(E,F)** Pie chart of the source of methylated mRNA in CaCO_2_ cell and sh-ADAR1 CaCO_2_ cell.

In this study, we found 1,835 clean o^8^G methylation peaks in CaCO_2_ cells and 592 clean o^8^G methylation peaks in sh-ADAR1 CaCO_2_ cells. We mapped up to 1,279 CaCO_2_ annotation genes and 402 sh-ADAR1 CaCO_2_ cellular annotation genes. Among them, 2,131 o^8^G modification peaks were found in CaCO_2_ cells and sh-ADAR1 CaCO_2_ cells, corresponding to 1,478 o^8^G modification genes (Figures 2A,B). We further investigated the distribution of o^8^G peaks on chromosomes using Circos software and found that the number and distribution of o^8^G peaks on each chromosome of CaCO_2_ cells and senescent CaCO_2_ cells differed, with the most significant differences between chromosomes 1 and 11 (Figure 2C).

Further analysis of the source of the o^8^G methylation peak revealed that the distribution of the o^8^G modification covered all regions of the mRNA but was enriched in the coding sequence region (CDS) (Figures 2D–F). However, the o^8^G peak in CaCO_2_ cells and the o^8^G peak in senescent CaCO_2_ cells exhibit different patterns. The number of o^8^G peaks in the start codon (start C) of normal CaCO_2_ cells (CaCO_2_: 19.46% vs. sh-ADAR1 CaCO_2_: 14.86%) and the o^8^G peaks in the 5′ untranslated region (5′ UTR) (CaCO_2_: 7.79% vs. sh-ADAR1 CaCO_2_: 6.59%) was higher than that of senescent cancer cells.

The number of o^8^G peaks in CDS region of normal CaCO_2_ cells (CaCO_2_: 50.14%, vs. sh-ADAR1 CaCO_2_: 53.89%) and the number of o^8^G peaks in stop codon (stop C) (CaCO_2_: 14.39% vs. sh-ADAR1 CaCO_2_) were decreased compared with senescent cancer cells. In the meantime, the number of o8G peaks in 3′ untranslated region (3′ UTR) of normal CaCO_2_ cells (CaCO_2_: 8.23% vs. sh-ADAR1 CaCO_2_: 9.46%) were also decreased compared with senescent cancer cells.

### o^8^G motif analysis and differential o^8^G methylation analysis

**FIGURE 3:**
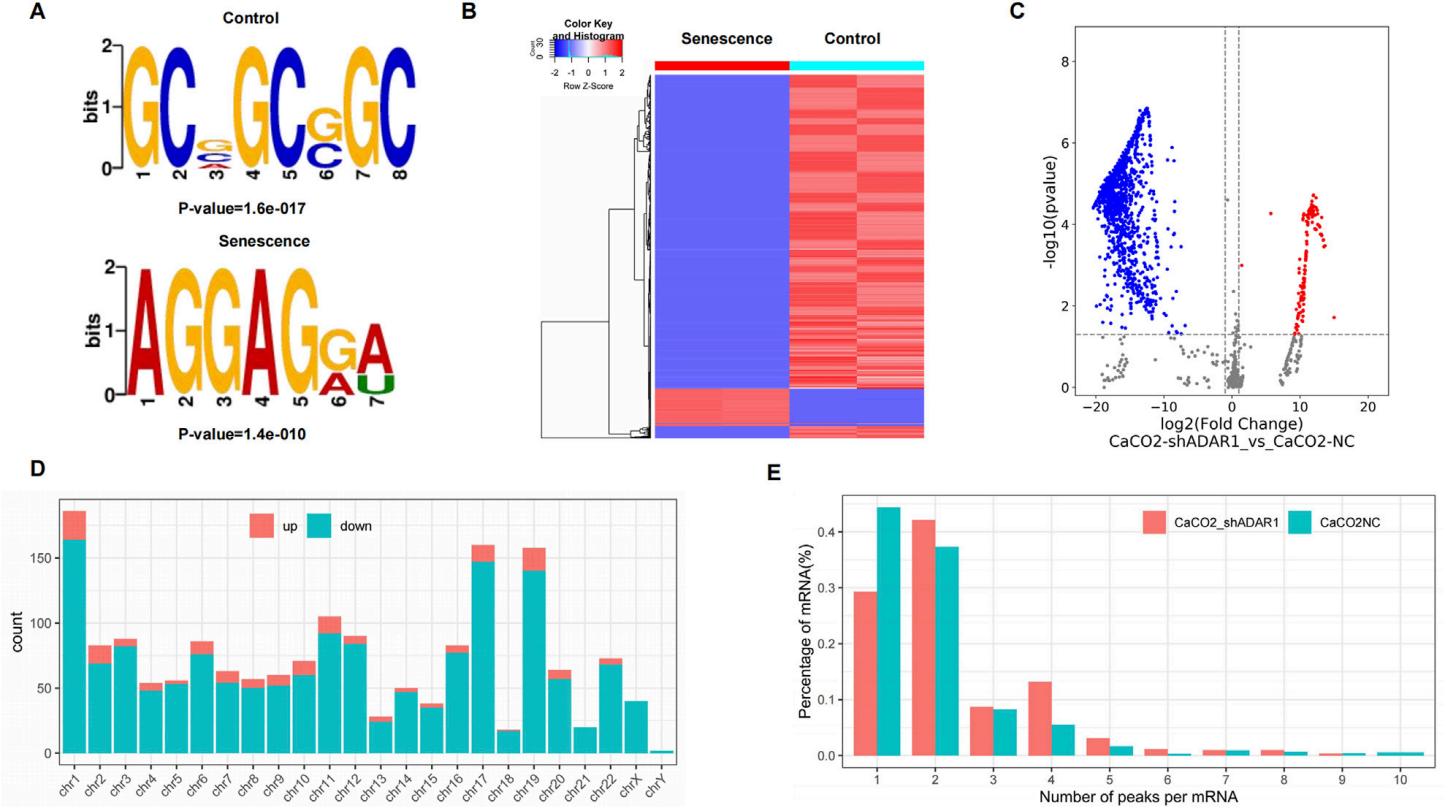
Characteristics of altered o^8^G peaks in CaCO_2_ cell and sh-ADAR1 CaCO_2_ cell. **(A)** The sequence motif of o^8^G sites in CaCO_2_ cell and sh-ADAR1 CaCO_2_ cell. **(B)** Cluster analysis of methylation in CaCO_2_ cell and sh-ADAR1 CaCO_2_ cell. **(C)** Volcano plots showing the significantly differential o^8^G peaks. **(D)** The distributions of altered o^8^G peaks in human chromosomes. **(E)** The number of o^8^G peaks in CaCO_2_ cell and sh-ADAR1 CaCO_2_ cell on each mRNA. Most mRNAs have only one methylation peak.

The results showed that GCWGCWGC was the most common and most confident methylation site motif in CaCO_2_ cells (p = 1.6e-017), while AGGAGWW is the most common and most confident methylation site motif in sh-ADAR1 CaCO_2_ cells (p = 1.8e-016) (Figure 3A). We performed methylation heat mapping and cluster analysis on the total data. The results of cluster analysis showed that there were significant differences in expression among the groups, and the expression was consistent within the groups. These differences are due to a decrease in the level of o^8^G modification due to aging (Figure 3B). In addition, we identified 142 upregulated o^8^G methylation peaks and 1,574 downregulated o^8^G methylation peaks of senescent CaCO_2_ compared to normal CaCO_2_ cells using DiffReps software (Figure 3C).

We have listed the top 10 mRNAs with the largest fold change in Tables 1, 2. Dysregulated o^8^G methylation peaks are distributed on all chromosomes, especially on chromosome 1 (Figure 3D). In addition, to determine the number of o^8^G peaks on each mRNA, we calculated the methylation peak and the mRNA corresponding to the methylation peak. The results showed that most of the mRNAs at the methylation site in sh-ADAR1 CaCO_2_ cells had only one methylation peak (29.8%), while CaCO_2_ cells had a higher proportion of methylation peaks (44.6%). At the same time, the number of mRNAs with three or more methylation peaks on 1 mRNA in sh-ADAR1 CaCO_2_ cells was higher than in CaCO_2_ cells (p < 0.05) (Figure 3E).

**TABLE 1:** Top 10 upregulated o8G methylation peaks.

Chrom	Peak ID	Gene name	Fold change	P-value
chr6	diffreps_peak_4602	DSP	13794.5	0.000426772
chr14	diffreps_peak_1645	CKB	12262.7	0.000379178
chr19	diffreps_peak_2638	BSG	11749.3	0.000294512
chr12	diffreps_peak_1153	SLC38A2	11508.7	0.000222202
chr10	diffreps_peak_723	DUSP5	8647.7	0.000190807
chr7	diffreps_peak_4918	SCRN1	7528.4	0.000114559
chr20	diffreps_peak_3483	SERINC3	6551.7	0.00010594
chr6	diffreps_peak_4845	AKAP12	6246.3	0.00010373
chr6	diffreps_peak_4847	AKAP12	5617.6	4.43843E-05
chr3	diffreps_peak_4059	ATP13A3	5613	0.000150712

**TABLE 2:** Top 10 downregulated o^8^G methylation peaks.

Chrom	Peak ID	Gene name	Fold change	P-value
chr22	diffreps_peak_3686	LIF	1222101	0.00116179
chr1	diffreps_peak_488	CAPN2	1152885.7	3.73521E-05
chr17	diffreps_peak_2358	HOXB9	1084237.4	0.003874857
chr17	diffreps_peak_2529	SLC16A3	1035492.8	3.31945E-05
chr11	diffreps_peak_895	AHNAK	1024423.2	6.46622E-05
chr11	diffreps_peak_817	PIK3C2A	969657.8	0.000253411
chr11	diffreps_peak_818	PIK3C2A	968524.1	0.000285916
chrX	diffreps_peak_5740	FLNA	936340.1	3.15226E-05
chr11	diffreps_peak_1066	ST14	933816.1	0.000764533
chr4	diffreps_peak_4177	CCNI	884241.4	5.97909E-05

### Gene ontology (GO) enrichment analysis

**FIGURE 4:**
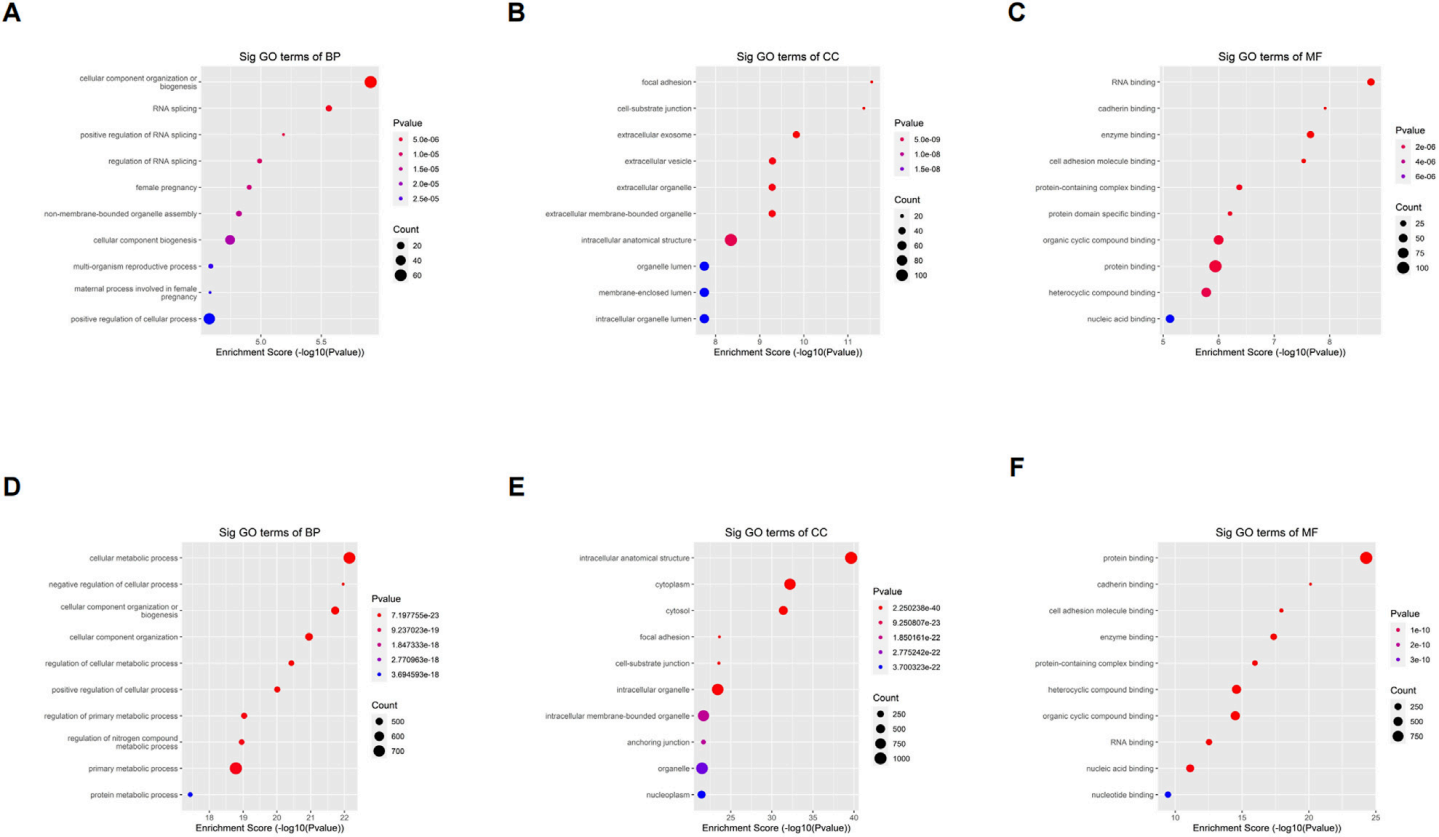
Gene ontology analyses of CaCO_2_ and shADAR1-CaCO_2_ cell. **(A)** Biological processes, **(B)** cellular components, and **(C)** molecular functions of genes annotated by upregulated o^8^G peaks in sh-ADAR1 CaCO_2_ cell group. **(D)** Biological processes, **(E)** cellular components, and **(F)** molecular functions of genes annotated by downregulated o^8^G peaks in sh-ADAR1 CaCO_2_ cell group. The top ten most significant terms are shown for each analysis.

To understand the biological function of differentially o^8^G-modified mRNA in sh-ADAR1 CaCO_2_ cells and CaCO_2_ cells, we performed GO analysis. For genes that are upregulated in o^8^G methylation in sh-ADAR1 CaCO_2_ cells, GO analysis showed that these genes are enriched in Cellular component organization or biogenesis (GO terminology: BP), Focal adhesion (GO term: CC), and RNA binding (GO term: MF) (Figures 4A–C). As for genes downregulated by o^8^G modification in sh-ADAR1 CaCO_2_ cells, GO analysis showed that these genes were mainly enriched in cellular metabolic process (GO term: BP), intracellular anatomical structure (GO term: CC), and protein binding (GO term: MF) (Figures 4D–F).

### Kyoto encyclopedia of genes and genomes (KEGG) pathway analysis

**FIGURE 5:**
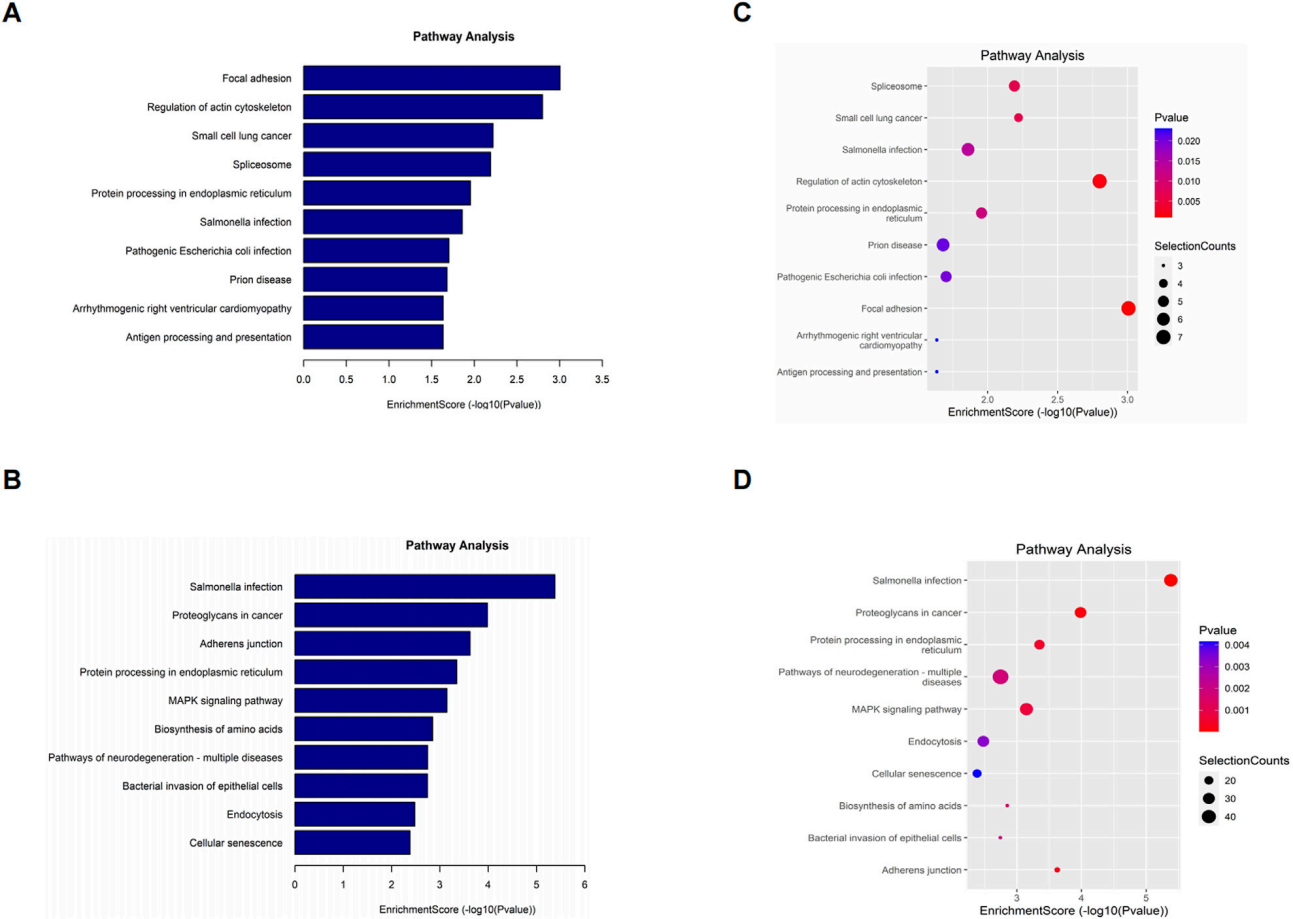
Kyoto Encyclopedia of Genes and Genomes analysis of differentially methylated genes in CaCO_2_ and shADAR1-CaCO_2_ cell. **(A,B)** Pathway analysis of up-methylated genes in sh-ADAR1 CaCO_2_ cell group. **(C,D)** Pathway analysis of down-methylated genes in sh-ADAR1 CaCO_2_ cell group. The top ten most significant terms are shown for each analysis.

To elucidate the biological function of differentially o^8^G-modified mRNAs in sh-ADAR1 CaCO_2_ cells and CaCO_2_ cells, we performed KEGG pathway analysis on differentially o^8^G-modified mRNAs. KEGG pathway analysis showed that the mRNA upregulated by o^8^G modification in sh-ADAR1 CaCO_2_ cells was enriched in Focal adhesion signaling pathway, Regulation of actin cytoskeleton signaling pathway and Small cell lung cancer signaling pathway (Figures 5A,B). mRNA downregulated by o8G was enriched in the *Salmonella* infection signaling pathway, Proteoglycans in cancer signaling pathway and Adherens junction signaling pathway (Figures 5C,D).

## Discussion

As an especially important post-transcriptional modification, RNA modification has been shown to be closely related to cell senescence and the occurrence and progression of cancer (Zhou et al., 2024; Cavinato et al., 2021), but there are few studies on RNA modification and cancer cell senescence. The o^8^G modification is an RNA modification produced by the attack of guanine in mRNA by reactive oxygen species in mammalian cells, which can pair with adenine and induce guanine-thymine (G > T) mutations, which is a modification in pathophysiology that causes disease phenotype through reactive oxygen species oxidation. o^8^G modifications in RNA can cause problems with abnormal quality and translation fidelity (Hahm et al., 2022). Due to the ability of o^8^G·A to base pair, o^8^G alters the structural and functional RNA-RNA interactions, enabling post-transcriptional regulation to be redirected (Seok et al., 2020). Recent studies have shown that tRF-1-AspGTC has o^8^G (5o^8^G) modification on the 5th G in its seed region, so that 5o^8^G tRF-1-AspGTC can retarget new downstream genes WNT5A and CASP (Zhang et al., 2024). At present, the mechanism of oxidative modification of o^8^G RNA, as a new class of RNA modification, is far from elucidated (Eom et al., 2023). In this study, we explored the potential role of o8G modification in senescent cancer cells.

First, our study showed that knockdown of ADAR1 can effectively induce senescence in CRC cells, and knockdown of ADAR1 increases SA-β-gal activity. Next, we used MeRIP-seq to sequence the relevant samples and characterize the o^8^G modification profile of mRNA in CaCO_2_ cells and senescent CaCO_2_ cells using bioinformatics methods. A total of 2,131 o^8^G modification peaks were found in CaCO_2_ cells and senescent CaCO_2_ cells, corresponding to 1,478 o^8^G modification genes. A total of 1,835 o^8^G methylation peaks were found in CaCO_2_ cells and 592 o^8^G methylation peaks were found in senescent CaCO_2_ cells. The number of mRNA o^8^G methylation peaks in CaCO_2_ cells were significantly higher than that in senescent CaCO_2_ cells. The results of cluster analysis showed that the degree of methylation could clearly distinguish CaCO_2_ cells from senescent CaCO_2_ cells, which further confirmed the potential relationship between o^8^G and senescent cancer cells. The o^8^G peaks were distributed in all regions of mRNA, and the CDS region had the highest proportion. The o^8^G peaks is also found on all chromosomes, mainly on chromosome 1, chromosome 17 and chromosome 19.

We annotated 1,279 genes with altered o^8^G methylation in CaCO_2_ cells and 402 genes with altered o^8^G methylation in senescent CaCO_2_ cells. The peak value of mRNA o^8^G modification in CaCO_2_ cells was significantly higher than that in senescent CaCO_2_ cells. In senescent CaCO_2_ cells and normal CaCO_2_ cells, the peaks of o^8^G modification on some mRNAs, such as LIF, CAPN2, and HOXB9, can be as high as million-fold.

The number of start codons and o^8^G modifications of 5′ UTR in CaCO_2_ cells was higher than that in senescent cancer cells. In the CDS region, the number of stop codons and o^8^G modifications of 3′ UTR was relatively lower than that of senescent cancer cells. The first codon for mRNA translation is called the start codon, and AUG is the most common start codon, which encodes the amino acid methionine (Met) in eukaryotes and formyl methionine (fMet) in prokaryotes. During protein synthesis, the tRNA recognizes the start codon AUG with the help of some initiation factors and initiates the translation of the mRNA. Considering that the stop codon also has o^8^G modification, it is reasonable to suspect that o^8^G modification plays an important regulatory role in mRNA translation. Further research is needed on how specific o^8^G modification sites regulate the translation of specific mRNAs. 3′ UTR plays a key role in post-transcriptional regulation, influencing gene expression and mRNA stability, localization, and the occurrence of post-transcriptional modifications. Depending on the properties of the 3′ UTR, o^8^G modification of the 3′ UTR may affect the binding of non-coding RNAs or RNA-binding proteins (RBPs) to mRNA, thereby regulating mRNA translation and stability.

We used the traditional method of predicting other RNA modifications, such as the m^5^C motif, to predict the possible motif of o^8^G modification, due to the lack of relevant studies on o^8^G, the o^8^G modification motif on the mRNA is still inconclusive, which depends on more sequencing to reveal the characteristics of the motif.

We found that differentially o^8^G-modified transcripts were enriched in a variety of important cell biology processes, such as Cellular component organization or biogenesis, Focal adhesion, and RNA binding, suggest that o^8^G modification may be involved in these biological processes regulating cancer cell base aging. Through KEGG analysis, we found that differentially o^8^G methylated mRNA was enriched in some key cancer-related pathways, such as Small cell lung cancer signaling pathway, Proteoglycans in cancer signaling pathway. It also proves from another aspect that o^8^G modification is closely related to the development of cancer.

In conclusion, our study shows that when cancer cells undergo senescence, their o^8^G modification spectrum changes significantly, and the changes are reflected in two aspects, one is the change in the number of modifications, and the other is the change in the distribution of modifications. We also performed further bioinformatics analysis of mRNA with changes in o^8^G modification and GO and KEGG analyses preliminarily revealed the potential direction of o^8^G modification on the regulation of senescent cancer cells. In follow-up studies, further in-depth analysis of the specific regulatory mechanism of o^8^G modification on cancer cell aging is expected to provide innovative ideas for the treatment of cancer.

## Data Availability

The datasets presented in this study can be found in online repositories. The names of the repository/repositories and accession number(s) can be found below: https://www.ncbi.nlm.nih.gov/geo/, GSE284992.

## Supplementary material

SUPPLEMENTARY FIGURE S1 β-Gal showed that CaCO_2_ cells senescent compared with normal cells after knockdown of ADAR1.

## Abbreviations

o^8^G, 8-oxoguanine; m^5^C, 5-methylcytosine; GO, Gene ontology; KEGG, Kyoto Encyclopedia of Genes and Genomes; BP, biological processes; MF, molecular functions; CC, cellular components; IP, immunoprecipitation.
