# Supplementary material for: Overview of distinct 8-oxoguanine profiles of messenger RNA in normal and senescent cancer cells
Source: Front Cell Dev Biol. 2025 Feb 28;13:1443888. doi: 10.3389/fcell.2025.1443888 (PMC11906479; doi:10.3389/fcell.2025.1443888)
Supplement: Supplementary file 1 [file DataSheet1.zip › Supplementary/Table S1.doc]

**Supplementary data for**

**Overview of distinct** **8-Oxoguanine profiles of messenger RNA in normal and senescence colorectal cancer cells**

**Supplementary table 1 Sequences for shRNA**

| **Name** | **Sequences (5’ – 3’)** |
| --- | --- |
| sh-ADAR1 | GCCCACTGTTATCTTCACTTT |
