# Supplementary material for: Overview of distinct 8-oxoguanine profiles of messenger RNA in normal and senescent cancer cells
Source: Front Cell Dev Biol. 2025 Feb 28;13:1443888. doi: 10.3389/fcell.2025.1443888 (PMC11906479; doi:10.3389/fcell.2025.1443888)
Supplement: Supplementary file 1 [file DataSheet1.zip › Supplementary/o8G-Figure legends.doc]

**Fig. 1 Flowchart of the study**

**Fig.2 Characteristics of o^8^G peaks in CaCO2 cell and sh-ADAR1 CaCO2 cell**

(A) Venn diagram of o^8^G peaks in CaCO2 cell and sh-ADAR1 CaCO2 cell.
(B) Venn diagram of o^8^G genes in CaCO2 cell and sh-ADAR1 CaCO2 cell.
(C) Visualization of o^8^G at the chromosome level in CaCO2 cell and sh-ADAR1 CaCO2 cell.
(D) Accumulation of the region of average o^8^G peaks along all transcripts in CaCO2 cell and sh-ADAR1 CaCO2 cell.

(E-F) Pie chart of the source of methylated mRNA in CaCO2 cell and sh-ADAR1 CaCO2 cell.

**Fig. 3** **Characteristics of altered o^8^G peaks in CaCO2 cell and sh-ADAR1 CaCO2 cell**

(A) The sequence motif of o^8^G sites in CaCO2 cell and sh-ADAR1 CaCO2 cell.
(B) Cluster analysis of methylation in CaCO2 cell and sh-ADAR1 CaCO2 cell.
(C) Volcano plots showing the significantly differential o^8^G peaks.
(D) The distributions of altered o^8^G peaks in human chromosomes.
(E) The number of o^8^G peaks in CaCO2 cell and sh-ADAR1 CaCO2 cell on each mRNA. Most mRNAs have only one methylation peak.

**Fig. 4 Gene ontology analyses of CaCO2 and shADAR1-CaCO2 cell**

(A) Biological processes, (B) cellular components, and (C) molecular functions of genes annotated by upregulated o^8^G peaks in sh-ADAR1 CaCO2 cell group.

(D) Biological processes, (E) cellular components, and (F) molecular functions of genes annotated by downregulated o^8^G peaks in sh-ADAR1 CaCO2 cell group.

The top ten most significant terms are shown for each analysis.

**Fig.5 Kyoto Encyclopedia of Genes and Genomes analysis of differentially methylated genes in CaCO2 and shADAR1-CaCO2 cell**

(A-B) Pathway analysis of up-methylated genes in sh-ADAR1 CaCO2 cell group.

(C-D) Pathway analysis of down-methylated genes in sh-ADAR1 CaCO2 cell group.

The top ten most significant terms are shown for each analysis.
