# Supplementary material for: Overview of distinct 8-oxoguanine profiles of messenger RNA in normal and senescent cancer cells
Source: Front Cell Dev Biol. 2025 Feb 28;13:1443888. doi: 10.3389/fcell.2025.1443888 (PMC11906479; doi:10.3389/fcell.2025.1443888)
Supplement: Supplementary file 1 [file DataSheet1.zip › Supplementary/Table.doc]

| **Chrom** | **Peak ID** | **Gene Name** | **Fold change** | **P-value** |
| --- | --- | --- | --- | --- |
| chr6 | diffreps_peak_4602 | DSP | 13794.5 | 0.000426772 |
| chr14 | diffreps_peak_1645 | CKB | 12262.7 | 0.000379178 |
| chr19 | diffreps_peak_2638 | BSG | 11749.3 | 0.000294512 |
| chr12 | diffreps_peak_1153 | SLC38A2 | 11508.7 | 0.000222202 |
| chr10 | diffreps_peak_723 | DUSP5 | 8647.7 | 0.000190807 |
| chr7 | diffreps_peak_4918 | SCRN1 | 7528.4 | 0.000114559 |
| chr20 | diffreps_peak_3483 | SERINC3 | 6551.7 | 0.00010594 |
| chr6 | diffreps_peak_4845 | AKAP12 | 6246.3 | 0.00010373 |
| chr6 | diffreps_peak_4847 | AKAP12 | 5617.6 | 4.43843E-05 |
| chr3 | diffreps_peak_4059 | ATP13A3 | 5613 | 0.000150712 |

**Table 1 Top Ten Up regulated o8G Methylation Peaks**

**Table 2 Top Ten Down regulated o8G Methylation Peaks**

| **Chrom** | **Peak ID** | **Gene Name** | **Fold change** | **P-value** |
| --- | --- | --- | --- | --- |
| chr22 | diffreps_peak_3686 | LIF | 1222101 | 0.00116179 |
| chr1 | diffreps_peak_488 | CAPN2 | 1152885.7 | 3.73521E-05 |
| chr17 | diffreps_peak_2358 | HOXB9 | 1084237.4 | 0.003874857 |
| chr17 | diffreps_peak_2529 | SLC16A3 | 1035492.8 | 3.31945E-05 |
| chr11 | diffreps_peak_895 | AHNAK | 1024423.2 | 6.46622E-05 |
| chr11 | diffreps_peak_817 | PIK3C2A | 969657.8 | 0.000253411 |
| chr11 | diffreps_peak_818 | PIK3C2A | 968524.1 | 0.000285916 |
| chrX | diffreps_peak_5740 | FLNA | 936340.1 | 3.15226E-05 |
| chr11 | diffreps_peak_1066 | ST14 | 933816.1 | 0.000764533 |
| chr4 | diffreps_peak_4177 | CCNI | 884241.4 | 5.97909E-05 |
